# Device‐Specific Factors Associated With Device‐Related Infection Prevention and Control Practices in Long‐Term Care Hospitals: A Multicenter Multilevel Study

**DOI:** 10.1155/jonm/8122821

**Published:** 2026-07-16

**Authors:** Soo Min Lee, Yeo Kyoung Kim

**Affiliations:** ^1^ Department of Nursing, College of Nursing, Dong-A University, Busan, South Korea, donga.ac.kr

**Keywords:** infection control, long-term care, mediation analysis, multilevel analysis, organizational culture, resource support

## Abstract

**Aim:**

To examine multilevel factors associated with Foley catheter– and central venous catheter (CVC)–related infection prevention and control (IPC) practices among nursing staff in long‐term care hospitals (LTCHs) and to test whether resource support mediates the relationship between organizational culture and IPC practices.

**Background:**

LTCHs are vulnerable to device‐related healthcare‐associated infections, yet evidence on how individual‐ and organizational‐level factors shape IPC practices across device types in these settings remains limited.

**Methods:**

In this multicenter cross‐sectional study, we surveyed 197 nursing staff with at least 6 months of clinical experience from 11 LTCHs with 150 or more beds in South Korea. Foley catheter and CVC IPC practice scores were the primary outcomes. Potential associated factors included IPC knowledge, job stress, organizational culture, and resource support. Linear mixed models with hospitals as a random effect were used to identify associated factors, and bootstrap mediation analysis (2000 replications) was used to test the indirect pathway from organizational culture through resource support to IPC practice.

**Results:**

Resource support was most strongly associated with both Foley (*β* = 0.334, *p* < 0.001) and CVC (*β* = 0.379, *p* < 0.001) practice scores. Organizational culture was significantly associated with Foley catheter IPC practice (*β* = 0.233, *p* = 0.007) but not CVC IPC practice (*p* = 0.292). Mediation analysis with resource support as the mediator revealed partial mediation for Foley catheter IPC practice (mediation ratio = 48.8%) and near‐complete mediation for CVC IPC practice (mediation ratio = 75.3%). IPC knowledge was significant only for CVC IPC practice (*β* = 0.198, *p* = 0.004), whereas job stress showed a suppressive effect, becoming significant in the multivariate model for both devices.

**Conclusion:**

The same mediation model operated through qualitatively different pathways depending on the device type. These findings suggest that a one‐size‐fits‐all approach may be insufficient to improve device‐related IPC practices in LTCHs.

**Implications for Nursing Management:**

Nurse managers in LTCHs should target different levers by device—securing structural and training readiness for CVC care, while reinforcing accountability norms and peer monitoring for Foley catheter care.

## 1. Background

Long‐term care hospitals (LTCHs) primarily care for older adults and patients with chronic conditions who require prolonged hospitalization. The combination of their immunocompromised status, functional dependence, and frequent exposure to invasive medical devices renders the patients in these settings particularly vulnerable to healthcare‐associated infections (HAIs). A surveillance study using the McGeer criteria in South Korean LTCHs reported an HAI incidence of 1.57 per 1000 patient‐days and 30.38 per 100 admitted patients [1]. Likewise, a European point prevalence survey reported a crude HAI prevalence of 3.7% in long‐term care facilities, where respiratory and urinary tract infections were the most common HAIs [2]. Given that HAIs contribute to increased antibiotic use, prolonged hospitalization, functional decline, and higher mortality, HAI prevention is critical to both patient safety and efficient resource use in LTCHs [2, 3].

Invasive medical devices represent a major source of HAIs. Central venous catheters (CVCs) and indwelling urinary (Foley) catheters are directly associated with central line–associated bloodstream infections (CLABSIs) and catheter‐associated urinary tract infections (CAUTIs), respectively. Previous surveillance studies have reported CLABSI rates of 0.81 and 2.71 infections per 1000 catheter‐days in Korean general wards and intensive care units, respectively [4], and CAUTI rates ranging from 0 to 7.3 infections per 1000 catheter‐days among U.S. long‐term care residents, with a mean of 3.2 infections per 1000 catheter‐days [5]. The economic burden is also substantial, with additional inpatient costs estimated at US$13,793 per CAUTI case and US $48,108 per CLABSI case [6]. These clinical and economic burdens underscore the need for standardized, evidence‐based infection prevention and control (IPC) measures, which have been shown to reduce device‐related infections in clinical practice [7, 8]. However, LTCHs often face constraints in infection prevention staffing, staff training, and IPC‐related infrastructure [9, 10]. In these settings, consistent device management is challenging and education‐only approaches are unlikely to be sustainable. Accordingly, establishing the level of IPC practices related to invasive devices and clarifying why practices vary across personnel and facilities are critical to design effective IPC strategies in LTCHs. In particular, since invasive devices require different types of care, practices should be examined separately for each device to inform appropriate strategies.

Multiple factors influence IPC practices related to invasive devices. At an individual level, knowledge and psychosocial factors may influence IPC practices. Job stress has been shown to predict turnover among nurses [11], and burnout and related workforce strain may compromise patient safety and contribute to worse HAI outcomes [12, 13]. A recent systematic review, largely in coronavirus‐related clinical contexts, suggested that excessive workload and heightened stress could negatively affect motivation and adherence to IPC practices [14]. However, most of the existing literature has focused primarily on individual‐level determinants, such as knowledge, attitudes, and stress, and many studies have been conducted within single institutions. This limits the ability to capture variations in IPC practices across facilities and to determine whether differences in environmental and institutional contexts contribute to practices beyond individual characteristics. Given that healthcare workers are embedded in institutions, IPC practices are inherently shaped by a hierarchical context. Accordingly, studies on IPC practices across multiple facilities should account for clustering and between‐hospital variations, making multilevel approaches appropriate [15].

At the organizational level, resource support, including the availability of essential materials, educational opportunities, and adequate staffing, aligns with the core components emphasized in the World Health Organization’s guidelines for IPC implementation [16]. Evidence from European hospitals suggests that organizational support is essential for effective IPC implementation [17]. In addition to resource support, organizational culture may influence IPC practices. Organizational culture reflects shared values and norms regarding infection prevention, and a positive safety culture has been linked to improved IPC processes and reduced HAIs [7, 18]. Evidence from acute‐care settings suggests that resource support and organizational culture are closely related to device‐related IPC practice among nurses [19, 20]. However, most of this evidence has been generated in acute‐care hospitals and intensive care units, as reflected in major guidelines for preventing CLABSIs and CAUTIs [21, 22], whereas evidence from LTCHs remains limited [23]. Moreover, although organizational factors are recognized as important, how they translate into IPC practices in LTCHs remains unclear. From an implementation perspective, identifying such mechanisms is important because it helps specify actionable levers for improvement. Building on the WHO’s emphasis on both “system change” and “culture change” as complementary components of multimodal IPC strategies [16], it is plausible that organizational culture contributes to IPC practice partly through its influence on the mobilization and availability of resource support.

In summary, the existing research provides limited guidance on how individual‐ and organizational‐level factors jointly shape device‐related IPC practices across heterogeneous LTCH environments. In particular, it remains unclear whether IPC practices are shaped by different factors related to the device type. To address these gaps, in this study, we used a multicenter, multilevel design to examine the factors associated with Foley catheter– and CVC‐related IPC practices among nursing staff in LTCHs and to test whether resource support mediates the relationship between organizational culture and IPC practice. Thus, in this study, we aimed to provide evidence for more targeted and feasible device‐specific IPC improvement strategies in LTCH settings.

## 2. Methods

### 2.1. Study Design

This was a multicenter, cross‐sectional survey of nursing staff in multiple LTCHs. We examined IPC practices related to CVCs and Foley catheters, identified individual‐ and organizational‐level factors associated with practices using multilevel analyses, and tested whether resource support mediated the association between organizational culture and IPC practices.

### 2.2. Setting and Participants

We recruited nursing staff with at least 6 months of clinical experience from 11 LTCHs with 150 or more beds in South Korea. Nurse managers who did not participate directly in patient care were excluded. The sample size was estimated using G∗Power (Version 3.1.9.7), with the following parameters set for multiple regression: significance level of 0.05, effect size of 0.17, power of 0.90, and 17 associated factors, yielding a minimum of 160 participants. Because a priori power analysis for linear mixed models (LMMs) requires assumptions about design parameters, such as the intraclass correlation coefficient and variance components, a formal LMM‐based sample size estimation was not feasible in advance [24]. Therefore, we used a multiple regression‐based power analysis as a pragmatic approximation. Accounting for an attrition rate of approximately 20%, we set the target enrollment at 200 participants.

### 2.3. Measurements

*IPC Knowledge*: IPC knowledge was measured using a 35‐item instrument originally developed by Ha et al. [25] for invasive device‐related IPC guidelines and subsequently revised by Kim [26]. The instrument comprises 17 Foley catheter and 18 CVC items, each scored as correct (1 point) or incorrect/unknown (0 points). Higher scores indicated greater knowledge. Reliability in the original study was Kuder–Richardson 20 (KR‐20) = 0.67 [26]. Reliability in this study was KR‐20 = 0.45 for CVC knowledge and KR‐20 = 0.62 for Foley knowledge.

*Job Stress*: Job stress related to infection control was assessed using a 32‐item instrument developed by Her and Kim [27] and revised by Jang [28]. Items were rated on a 5‐point Likert scale (total range: 32–160), with higher scores indicating greater stress. Reliability in the original study was Cronbach’s *α* = 0.95 [28]. Reliability in this study was Cronbach’s *α* = 0.98.

*Organizational Culture*: Organizational safety culture for IPC was measured using a 10‐item instrument developed by Park [29] and adapted for IPC by Moon [30]. Items were rated on a 7‐point Likert scale (total range: 10–70). Higher scores indicated a more positive IPC organizational culture. Reliability in the original study was Cronbach’s *α* = 0.85 [30]. Reliability in this study was Cronbach’s *α* = 0.88.

*Resource Support*: Resource support for invasive device‐related HAI prevention was evaluated using an 11‐item instrument developed by Moon and Jang [20]. The items are rated on a 5‐point Likert scale, with higher scores reflecting greater resource availability. The content validity index was 0.94 and Cronbach’s *α* = 0.92 in the original study. Reliability in this study was Cronbach’s *α* = 0.92.

*IPC Practice*: IPC practice was measured using a 32‐item instrument developed by Ha et al. [25] and revised by Kim [26], comprising 17 Foley catheter and 15 CVC items. Items were rated on a 5‐point Likert scale (Foley range: 17–85; CVC range: 15–75), with higher scores indicating better practice. The Foley and CVC IPC practice scores were analyzed separately. Reliability in the original study was Cronbach’s *α* = 0.84 [26]. Reliability in this study was Cronbach’s *α* = 0.86 for CVC IPC practice and Cronbach’s *α* = 0.76 for Foley catheter IPC practice.

### 2.4. Data Collection

Data were collected between January and July 2025 following approval from the Institutional Review Board of Dong‐A University (Approval No. 2‐1040709‐AB‐N‐01‐202410‐HR‐048‐02). Approximately 50 LTCHs with 150 or more beds in the study region were invited to participate, and 11 expressed willingness to cooperate. Because this multilevel study involved both facility‐level data collection and individual‐level survey data from nursing staff nested within each facility, we first contacted hospitals and to obtain institutional permission and cooperation for data collection. After a hospital agreed to cooperate, recruitment of eligible nursing staff was initiated within that hospital. Following an overview of the study, online survey links with QR codes were distributed to the nursing departments. Individual participation was voluntary and anonymous, and participants provided online informed consent after reading the study information and before completing the questionnaire. A total of 200 responses were collected. After excluding three with insufficient responses, 197 were included in the final analysis.

### 2.5. Statistical Analysis

Data were analyzed using IBM SPSS Statistics software (Version 25.0) and the lme4 package in R. Descriptive statistics were calculated for the demographic characteristics and key study variables. Differences in IPC practice scores by demographic characteristics were examined using *t*‐tests, analysis of variance, and Fisher’s exact test, with Scheffé’s post hoc tests where applicable. Bivariate associations between the study variables were assessed using Pearson’s correlation coefficients.

To identify the factors associated with IPC practices, while accounting for the hierarchical data structure (individuals nested within hospitals), we fitted LMMs with hospitals as random effects. In the Foley model, age, job category, and education, identified as significant in the univariate analysis, were included as covariates. In the CVC model, job category was included. To test whether resource support mediates the relationship between organizational culture and IPC practices, we conducted a mediation analysis using bootstrapping with 2000 replications. The mediation was considered significant when the 95% confidence interval (CI) for the indirect effect did not reach zero. Statistical significance was set at *p* < 0.05. For the sensitivity analysis, we additionally adjusted for facility‐level IPC capacity using the WHO Infection Prevention and Control Assessment Framework (IPCAF) total score [31] (Supporting Table S1 in Additional File 1).

## 3. Results

### 3.1. Participant Characteristics

Of the 197 participants, 186 (94.4%) were female (Table 1). The largest age group was 50–59 years old (*n* = 65, 33.0%), and the majority of participants has completed a college or university degree (*n* = 141, 71.6%). Registered nurses comprised 65.5% (*n* = 129), followed by nursing assistants (*n* = 57, 28.9%) and care workers (*n* = 11, 5.6%). Approximately one‐third (*n* = 66, 33.5%) had 15 or more years of clinical experience, and 185 (93.9%) had received IPC education.

**TABLE 1:** Participant characteristics and IPC practice scores by demographic variables.

Variable	Category	*n* (%)	Foley IPC practice		CVC IPC practice	
			M ± SD	*p* (Scheffé)	M ± SD	*p* (Scheffé)
Age	< 40 yr^a^	58 (29.4)	63.62 ± 9.20	0.012	61.45 ± 11.25	0.138
	40–49 yr^b^	50 (25.4)	63.40 ± 9.12	a,b,c < d	61.10 ± 10.27	
	50–59 yr^c^	65 (33.0)	67.11 ± 7.92		64.08 ± 8.69	
	≥ 60 yr^d^	24 (12.2)	68.96 ± 9.87		65.67 ± 9.10	

Sex	Female	186 (94.4)	65.22 ± 9.11	0.356	62.76 ± 9.91	0.898
	Male	11 (5.6)	67.82 ± 7.93		62.36 ± 12.27	

Education	≤ Middle school^a^	3 (1.5)	77.67 ± 12.70	0.036	70.67 ± 7.51	0.153
	≤ High school^b^	34 (17.3)	67.50 ± 7.93	b,c,d < a	65.35 ± 8.75	
	≤ College/university^c^	141 (71.6)	64.76 ± 9.13		61.88 ± 10.30	
	≥ Graduate school^d^	19 (9.6)	64.11 ± 8.45		63.21 ± 9.60	

Job category	Care worker^a^	11 (5.6)	70.27 ± 12.38	0.004	65.73 ± 9.12	0.036
	Nursing assistant^b^	57 (28.9)	67.82 ± 7.33	c < a,b	65.18 ± 8.57	c < a,b
	Registered nurse^c^	129 (65.5)	63.86 ± 9.10		61.41 ± 10.48	

Experience	< 5 yr	42 (21.3)	68.05 ± 7.24	0.108	64.14 ± 10.66	0.499
	5–< 10 yr	41 (20.8)	65.80 ± 8.66		63.83 ± 8.21	
	10–< 15 yr	48 (24.4)	64.88 ± 11.01		62.25 ± 11.55	
	≥ 15 yr	66 (33.5)	63.74 ± 8.50		61.53 ± 9.45	

IPC education	No	12 (6.1)	62.00 ± 10.87	0.184	59.33 ± 14.28	0.225
	Yes	185 (93.9)	65.58 ± 8.91		62.96 ± 9.69	

*Note:* Post hoc comparisons conducted using the Scheffé test. Superscripts (a, b, c, d) indicate subgroups; significant differences are noted in the *p* column.

Abbreviation: M ± SD = mean ± standard deviation.

### 3.2. IPC Practice Scores by Demographic Characteristics

The overall mean Foley catheter IPC practice score was 65.37 ± 9.05, and the overall mean CVC IPC practice score was 62.74 ± 10.02. The Foley catheter IPC practice scores differed significantly by age (*p* = 0.012), education level (*p* = 0.036), and job category (*p* = 0.004), whereas no significant differences were observed for sex, years of experience, or IPC education status. The CVC IPC practice scores differed significantly only by job category (*p* = 0.036). Post hoc comparisons using Scheffé’s test revealed that registered nurses scored significantly lower than care workers and nursing assistants for Foley and CVC IPC practices (Table 1).

### 3.3. Bivariate Correlations Among Variables

Organizational culture was significantly and positively correlated with both Foley (*r* = 0.439, *p* < 0.001) and CVC (*r* = 0.328, *p* < 0.001) practice scores, and resource support showed the strongest correlation with both Foley (*r* = 0.472, *p* < 0.001) and CVC (*r* = 0.432, *p* < 0.001) practice scores (Table 2). CVC IPC knowledge was weakly but significantly correlated with CVC IPC practice (*r* = 0.141, *p* = 0.048), whereas Foley IPC knowledge showed no significant correlation with Foley catheter IPC practice (*p* = 0.143). Job stress was not significantly correlated with either the Foley (*p* = 0.114) or CVC (*p* = 0.246) practice scores.

**TABLE 2:** Descriptive statistics and bivariate correlations among variables.

Variable	M ± SD	Foley IPC practice		CVC IPC practice	
		*r*	*p*	*r*	*p*
Job stress	108.29 ± 25.59	0.113	0.114	0.083	0.246
Organizational culture	53.52 ± 9.64	0.439	< 0.001	0.328	< 0.001
Resource support	40.46 ± 8.75	0.472	< 0.001	0.432	< 0.001
IPC knowledge (Foley)	13.48 ± 2.18	−0.105	0.143	−0.009	0.898
IPC knowledge (CVC)	12.90 ± 2.19	−0.034	0.640	0.141	0.048
IPC practice (Foley)	65.37 ± 9.05	—		0.622	< 0.001
IPC practice (CVC)	62.74 ± 10.02	0.622	< 0.001	—	

*Note: r* = Pearson’s correlation coefficient.

### 3.4. Factors Associated With IPC Practice: LMMs

After accounting for hospital‐level clustering and relevant covariates, resource support emerged as the strongest factor associated with Foley (*β* = 0.334, *p* < 0.001) and CVC (*β* = 0.379, *p* < 0.001) practice scores (Table 3). Organizational culture (*β* = 0.233, *p* = 0.007) and job stress (*β* = 0.185, *p* = 0.005) were also significantly and positively associated with Foley catheter IPC practice, whereas IPC knowledge was not (*p* = 0.643). Job stress (*β* = 0.144, *p* = 0.029) and IPC knowledge (*β* = 0.198, *p* = 0.004) were significantly and positively associated with CVC IPC practice, but organizational culture was not (*β* = 0.091, *p* = 0.292).

**TABLE 3:** Factors associated with IPC practice: linear mixed model results.

Variable	Foley IPC practice					CVC IPC practice				
	*B*	SE	*β*	*t*	*p*	*B*	SE	*β*	*t*	*p*
Resource support	0.346	0.095	0.334	3.656	< 0.001	0.434	0.103	0.379	4.200	< 0.001
Organizational culture	0.219	0.081	0.233	2.705	0.007	0.095	0.090	0.091	1.057	0.292
Job stress	0.065	0.023	0.185	2.848	0.005	0.057	0.026	0.144	2.199	0.029
IPC knowledge	0.149	0.321	0.036	0.464	0.643	0.906	0.315	0.198	2.880	0.004

*Note:* Foley model: adjusted for age, job category, and education level. CVC model: adjusted for job category. *B* = unstandardized regression coefficient; *β* = standardized regression coefficient.

Abbreviation: SE = standard error.

In the sensitivity analysis additionally adjusted for facility‐level IPC capacity, as measured by the IPCAF score, the direction, magnitude, and statistical significance of the primary fixed effects remained substantively unchanged. This finding supports the robustness of the main results to additional adjustment for facility‐level IPC capacity.

### 3.5. Mediation Analysis: Organizational Culture ⟶ Resource Support ⟶ IPC Practice

Bootstrap mediation analysis revealed that the indirect effect of organizational culture on Foley catheter IPC practices through resource support was significant (*B* = 0.184, 95% CI [0.082, 0.309]), as was the direct effect (*B* = 0.203, 95% CI [0.029, 0.359]), indicating partial mediation with a mediation ratio of 48.8% (Table 4). For CVC IPC practice, the direct effect was not significant (*B* = 0.093, 95% CI [−0.074, 0.280]), whereas the indirect effect through resource support was significant (*B* = 0.220, 95% CI [0.111, 0.340]), yielding a mediation ratio of 75.3% and indicating near‐complete mediation.

**TABLE 4:** Mediation analysis results.

Effect	Foley IPC practice		CVC IPC practice	
	*B*	95% CI	*B*	95% CI
Direct effect	0.203	[0.029, 0.359]	0.093	[−0.074, 0.280]
Indirect effect	0.184	[0.082, 0.309]	0.220	[0.111, 0.340]
Mediation ratio	48.8%		75.3%	

*Note:* X = organizational culture; M = resource support; Y = IPC practice (CVC‐related/Foley catheter–related). Indirect effects were estimated using bootstrapping (2000 resamples) with 95% confidence intervals.

## 4. Discussion

In this multicenter, multilevel study of nursing staff in LTCHs, device‐related IPC practices were associated with factors operating at the individual and organizational levels. A key finding was that Foley catheter– and CVC‐related IPC practices were grounded in shared organizational conditions, but these conditions influenced IPC practice through different pathways depending on the device. These findings suggest that IPC improvement in LTCHs should move beyond a one‐size‐fits‐all approach and instead pursue context‐sensitive, device‐specific strategies aligned with institutional conditions.

Our mediation analyses suggest that organizational culture may influence IPC practices, partly through its effect on resource support. Previous studies have identified both organizational culture and resource support as important correlates of IPC practice [19, 20]. Our findings extend this literature by specifying a potential indirect pathway. For CVC‐related IPC practice, this indirect pathway was particularly evident. This pattern aligns with the technical demands of CVC bundle care. Core CVC practices require immediate access to dedicated supplies and high‐level procedural training [21]. Under such conditions, it may be difficult for cultural expectations to be reflected in routine practice when operational support is insufficient. This finding contrasts with results from Kim and Choi [19], who reported that organizational culture was the strongest factor associated with CLABSI prevention practice among nurses in tertiary acute‐care hospitals. The difference may reflect the distinct resource contexts of the two settings: In tertiary hospitals, resource availability is relatively assured, which may allow organizational culture to be more readily reflected in IPC practice. In long‐term care settings, however, where dedicated IPC supplies and trained personnel may be structurally inadequate [9, 10], resource support appears to link organizational culture to IPC practices. This finding suggests that the pathway from culture to practice is context‐dependent and that strengthening resource support (e.g., dressing carts, device‐specific training, and staffing) may be a prerequisite for improving CVC‐related IPC practices.

Foley catheter–related IPC practices showed both a direct association with organizational culture and an indirect pathway through resource support, indicating partial mediation. This pattern is consistent with the view that CAUTI prevention requires both technical and socioadaptive components [22]. Because Foley catheter care is embedded in daily practice and often involves multiple providers, organizational culture may support shared norms, communication, and routine reinforcement. Evidence from the AHRQ Safety Program for Long‐Term Care, involving more than 400 U.S. nursing homes, also showed that CAUTI rates declined after implementation of a multicomponent program addressing both technical and socioadaptive components [8]. In our study, this partial mediation pattern suggests that resource availability remains foundational, while organizational culture may also contribute to Foley catheter–related IPC practice through normative and relational processes, including shared norm and routine reinforcement. Collectively, these findings suggest that Foley catheter–focused improvement strategies should combine adequate resource provision with efforts to strengthen shared norms and accountability.

Resource support exhibited the most consistent association with IPC practices across both device domains. This finding is consistent with previous evidence linking resource support to IPC guideline performance [20]. This also aligns with qualitative evidence from European hospitals, identifying resource support as a key theme for successful IPC implementation [17]. A needs assessment of long‐term care facilities in Florida also reported gaps in dedicated IPC personnel and training resources [10]. The strong role of resource support may reflect infrastructural constraints in LTCH settings. Although evidence from LTCHs is lacking, they often have limited dedicated IPC staffing and material support, and the implementation of core IPC components may be incomplete [9, 10, 23]. Therefore, resource availability may have a particularly visible effect on practice, because these elements are more likely to be unmet in LTCHs. Improvements in IPC practice in LTCHs must prioritize adequate resource support and human capital as fundamental elements. The robustness of this finding was further supported by the sensitivity analyses additionally adjusted for facility‐level IPC capacity, in which the overall pattern and interpretation of the primary fixed effects remained substantially unchanged (Supporting Table S1 in Additional file 1).

IPC knowledge showed a more significant association with CVC‐related IPC practice than with Foley catheter–related IPC practice. These differences may reflect the distinct competencies required for each practice. In LTCHs, Foley catheter care often involves repeated daily care and routine procedures [32]. In such settings, differences in individual knowledge may not be clearly reflected in IPC practice. In contrast, CVC management may occur less frequently and be more procedurally complex, requiring precise knowledge of aseptic techniques and evidence‐based guidelines. Therefore, knowledge may be an important factor in IPC practice when procedural familiarity is limited. Although the internal consistency of the CVC knowledge subscale was low, this limitation may have attenuated rather than inflated the observed association. These findings suggest that efforts to improve CVC‐related IPC practice should emphasize device‐specific training to improve competency. Notably, this pattern differs from prior literature which suggested that knowledge is relevant to CAUTI‐related practice [33, 34]. For example, systematic reviews have emphasized healthcare workers’ knowledge as an important factor in CAUTI‐related practice [33, 34]. Differences in study settings may partly explain this discrepancy. Acute‐care hospitals and intensive care units generally have a stronger infrastructure, but the frequent turnover and continuous inflow of novice nurses may make knowledge a more salient factor of practice in those settings. In contrast, staffing in LTCHs may rely more on experienced personnel, while resources remain limited. Under such conditions, the marginal effect of knowledge may be attenuated because resource availability and workflow feasibility become primary bottlenecks.

Taken together, these findings indicate that improving invasive device‐related IPC in LTCHs requires device‐specific strategies and a stronger institutional infrastructure. A common infrastructure should first be established across devices, including standardized procedures, documentation systems, reliably supplied materials, and leadership support. On this foundation, interventions should be tailored according to the device type. For CVC‐related care, improving resource support and providing targeted training to strengthen care competencies may be key implementation priorities. In addition, for Foley catheter–related care, interventions should promote norm formation, structured peer monitoring, and the routinization of key procedures.

The findings regarding job stress require cautious interpretation. Although job stress showed no significant zero‐order correlation with IPC practice, it emerged as a significant positive factor in the adjusted mixed‐effects models. This pattern is inconsistent with previous literature, which typically reported that job stress and burnout are associated with reduced patient safety and higher HAI risk [12, 13]. For example, Cimiotti et al. [12] found that higher burnout was associated with significantly increased HAI rates in acute‐care hospitals, and a recent meta‐analysis by Li et al. [13] confirmed that nurse’s burnout was associated with lower patient safety performance. This discrepancy may reflect a statistical suppression effect [35], in which the unique association between job stress and IPC practice becomes apparent only after accounting for the shared variance with organizational culture and resource support. Within this context, two interpretations may explain the observed patterns in LTCHs. First, a positive association may reflect challenge stress rather than hindrance [36]. In resource‐constrained LTCH settings, staff members who are more closely involved in complex device management may experience greater job‐related tension because of their professional responsibilities. In this context, a higher IPC performance may be accompanied by greater psychological pressure. However, we cannot exclude the possibility that participants under high occupational pressure may have overestimated their adherence to self‐reported measures to meet perceived professional expectations [37]. Given these complexities, future studies should employ more refined measures to distinguish between challenge and hindrance stresses.

### 4.1. Limitations

This study had several limitations. First, its cross‐sectional design precludes causal inferences regarding the observed relationships and mediation pathways. Second, key constructs were measured by self‐reporting, which may have introduced a reporting bias. Third, internal consistency of the CVC‐specific knowledge subscale was low. This may partly reflect the fact that knowledge reflected by one item does not necessarily covary with that reflected by other items, which can further reduce internal consistency when knowledge is measured using multiple dichotomous questions [38]. Accordingly, the association between CVC knowledge and CVC‐related IPC practice should be interpreted cautiously; however, the low reliability may have attenuated the estimated association, suggesting that the true effect of knowledge on practice might actually be stronger than statistically observed. Finally, the number of participating hospitals was limited, which may have reduced the precision of between‐hospital variance estimates. Although random intercepts partially addressed clustering, larger multicenter studies will be needed to improve precision and generalizability. Future studies should examine device‐specific mechanisms in greater detail and test whether tailoring implementation strategies according to device type will lead to sustained improvements in IPC practices.

## 5. Conclusions

In this multicenter, multilevel study, device‐related IPC practices in LTCHs reflected both shared institutional conditions and device‐specific mechanisms. Resource support was a consistent determinant across both CVC‐ and Foley catheter–related IPC practices, whereas the role of organizational culture differed according to device type. These results indicate that uniform IPC strategies may not optimize practices across diverse invasive devices in LTCHs. For CVC‐related care, structural readiness and competency‐based training should be prioritized, whereas Foley catheter–related care may also benefit from shared norms, peer monitoring, and reinforcement of routine practice.

## Author Contributions

Soo Min Lee: conceptualization, supervision, investigation, data analysis, writing–original draft and review and editing, and project administration. Yeo Kyoung Kim: conceptualization, investigation and writing–original draft and review and editing.

## Funding

This work was supported by the National Research Foundation of Korea (NRF) grant funded by the Korean Government (MSIT) (grant number NRF‐2020R1G1A1102533).

## Disclosure

This funding source had no role in the study design, analysis, data interpretation, or decision to submit the manuscript for publication.

## Ethics Statement

This study was conducted in accordance with the Declaration of Helsinki. This study was approved by the Institutional Review Board of Dong‐A University (Approval No. 2‐1040709‐AB‐N‐01‐202410‐HR‐048‐02). All participants provided informed consent after reviewing the study information, which described the study objectives, procedures, voluntary nature of participation, protection of anonymity, and data management.

## Conflicts of Interest

The authors declare no conflicts of interest.

## Supporting Information

Additional supporting information can be found online in the Supporting Information section.

## Supporting information


**Supporting Information 1** Supporting Methods. Supporting Table S1. Linear mixed models additionally adjusted for facility‐level IPC capacity (IPCAF total score). Supporting References.

**Supporting Information 2** STROBE_checklist.

## Data Availability

The data collected and analyzed during the current study are available from the corresponding author upon reasonable request.
