# Supplementary material for: Device‐Specific Factors Associated With Device‐Related Infection Prevention and Control Practices in Long‐Term Care Hospitals: A Multicenter Multilevel Study
Source: J Nurs Manag. 2026 Jul 16;2026:8122821. doi: 10.1155/jonm/8122821 (PMC13374110; doi:10.1155/jonm/8122821)
Supplement: Supplementary file 1 — Supporting Information 1 Supporting Methods. Supporting Table S1. Linear mixed models additionally adjusted for facility‐level IPC capacity (IPCAF total score). Supporting References. [file JONM-2026-8122821-s001.docx]

**Supplementary Material**

**Supplementary Methods**

Facility-level IPC capacity was assessed using the World Health Organization (WHO) Infection Prevention and Control Assessment Framework (IPCAF). IPCAF is a standardized facility self-assessment tool that evaluates the implementation of IPC core components across eight domains and yields a total score ranging from 0 to 800, with higher scores indicating greater IPC capacity (WHO, 2018). In this study, the IPCAF total score was included only in sensitivity analyses to examine the robustness of the primary mixed-model estimates.

**Supplementary Table S1. Linear mixed models additionally adjusted for facility-level IPC capacity (IPCAF total score)**

| **Variable** | **Foley IPC practice** | | | | | **CVC IPC practice** | | | | |
| --- | --- | --- | --- | --- | --- | --- | --- | --- | --- | --- |
|  | **B** | **SE** | **β** | **t** | ***p*** | **B** | **SE** | **β** | **t** | ***p*** |
| Resource support | .351 | .095 | .339 | 3.678 | <.001 | .429 | .104 | .374 | 4.135 | <.001 |
| Organizational culture | .217 | .081 | .232 | 2.683 | .008 | .097 | .090 | .093 | 1.080 | .281 |
| Job stress | .065 | .023 | .183 | 2.813 | .005 | .056 | .026 | .142 | 2.162 | .032 |
| IPC knowledge | .171 | .326 | .041 | .525 | .600 | .899 | .315 | .196 | 2.853 | .005 |
| IPCAF total score | -.002 | .010 | -.021 | -.225 | .829 | .008 | .009 | .062 | .810 | .456 |

Note. Models accounted for clustering by hospital using random intercepts. The Foley model adjusted for age group, job category, and prior IPC education; the CVC model adjusted for job category. IPCAF was included as a fixed effect using the facility total score.

**Supplementary References**

World Health Organization. (2018). Infection prevention and control assessment framework at the facility level (IPCAF). WHO.
