# Supplementary material for: Device‐Specific Factors Associated With Device‐Related Infection Prevention and Control Practices in Long‐Term Care Hospitals: A Multicenter Multilevel Study
Source: J Nurs Manag. 2026 Jul 16;2026:8122821. doi: 10.1155/jonm/8122821 (PMC13374110; doi:10.1155/jonm/8122821)
Supplement: Supplementary file 2 — Supporting Information 2 STROBE_checklist. [file JONM-2026-8122821-s002.pdf]

STROBE Statement—checklist of items that should be included in reports of observational studies

	Item No.	Recommendation	Page No.	Relevant text from manuscript
**Title and abstract**	1	(*a*) Indicate the study's design with a commonly used term in the title or the abstract	2	Methods: In this multicenter cross-sectional study, we surveyed 197 nursing staff …
		(*b*) Provide in the abstract an informative and balanced summary of what was done and what was found	2-3	The abstract provides structured Background, Methods, Results, and Conclusion sections summarizing what was done and what was found.
**Introduction**				
Background/rationale	2	Explain the scientific background and rationale for the investigation being reported	4-6	The Background section explains the burden of device-related healthcare-associated infections in LTCHs, gaps in prior literature, and the need for device-specific multilevel evidence.
Objectives	3	State specific objectives, including any prespecified hypotheses	6	we used a multicenter, multilevel design to examine the factors associated with Foley catheter- and CVC-related IPC practices … and to test whether resource support mediates the relationship between organizational culture and IPC practice.
**Methods**				
Study design	4	Present key elements of study design early in the paper	7	This was a multicenter, cross-sectional survey of nursing staff in multiple LTCHs.
Setting	5	Describe the setting, locations, and relevant dates, including periods of recruitment, exposure, follow-up, and data collection	7, 9	• We recruited nursing staff with at least 6 months of clinical experience from 11 LTCHs with 150 or more beds in South Korea.<br>• Data were collected between January and July 2025 … Approximately 50 LTCHs … were invited … and 11 consented.
Participants	6	(*a*) *Cohort study*—Give the eligibility criteria, and the sources and methods of selection of participants. Describe methods of follow-up	7, 9	Eligibility and selection are described: nursing staff with at least 6 months of clinical experience were recruited; nurse managers not directly involved

		Case-control study—Give the eligibility criteria, and the sources and methods of case ascertainment and control selection. Give the rationale for the choice of cases and controls<br>*Cross-sectional study*—Give the eligibility criteria, and the sources and methods of selection of participants		in patient care were excluded; 200 responses were collected and 197 analyzed.
		(*b*) *Cohort study*—For matched studies, give matching criteria and number of exposed and unexposed<br>*Case-control study*—For matched studies, give matching criteria and the number of controls per case	N/A	Not applicable.
Variables	7	Clearly define all outcomes, exposures, predictors, potential confounders, and effect modifiers. Give diagnostic criteria, if applicable	7-10	Outcomes and candidate predictors are defined in Method section. Potential associated factors included IPC knowledge, job stress, organizational culture, and resource support; covariates are specified in the LMMs.
Data sources/ measurement	8*	For each variable of interest, give sources of data and details of methods of assessment (measurement). Describe comparability of assessment methods if there is more than one group	7-9	Each study variable is linked to its measurement instrument, number of items, response scale, score range, and reliability.
Bias	9	Describe any efforts to address potential sources of bias	18-19	Potential bias is acknowledged mainly in the Limitations.
Study size	10	Explain how the study size was arrived at	7	The sample size was estimated using G*Power … yielding a minimum of 160 participants … Accounting for an attrition rate of approximately 20%, we set the target enrollment at 200 participants.

Continued on next page

Quantitative variables	11	Explain how quantitative variables were handled in the analyses. If applicable, describe which groupings were chosen and why	7-8	.
Statistical methods	12	(*a*) Describe all statistical methods, including those used to control for confounding	9-10	Statistical methods included descriptive statistics, t-tests, analysis of variance, Fisher's exact test, Pearson correlations, linear mixed models with hospitals as random effects, and bootstrap mediation analysis with 2,000 replications.
		(*b*) Describe any methods used to examine subgroups and interactions	9-10	No formal subgroup or interaction analysis was reported. Device-specific analyses were performed separately for Foley catheter and CVC IPC practices, and mediation analysis tested an indirect pathway through resource support.
		(*c*) Explain how missing data were addressed	9	Missing questionnaires were handled by exclusion before analysis: After excluding three with insufficient responses, 197 were included in the final analysis.
		(*d*) *Cohort study*—If applicable, explain how loss to follow-up was addressed<br>*Case-control study*—If applicable, explain how matching of cases and controls was addressed<br>*Cross-sectional study*—If applicable, describe analytical methods taking account of sampling strategy	N/A	Not applicable.
		(*e*) Describe any sensitivity analyses	10, 16	• For the sensitivity analysis, we additionally adjusted for facility-level IPC capacity …<br>• Discussion also states that the overall pattern remained substantially unchanged (Supplementary Table S1).
**Results**				
Participants	13*	(a) Report numbers of individuals at each stage of study—eg numbers potentially eligible, examined for eligibility, confirmed eligible, included in the study, completing follow-up, and analysed	9-10	Participant flow is reported numerically: about 50 hospitals were invited; 11 consented; 200 responses were collected; 3 were excluded for insufficient responses; 197 were analyzed.
		(b) Give reasons for non-participation at each stage	9	Non-participation/ineligibility is partly reported: 3 responses were excluded because of insufficient responses. Reasons for hospital or participant non-participation were not detailed.

		(c) Consider use of a flow diagram	N/A	No flow diagram was provided.
Descriptive data	14*	(a) Give characteristics of study participants (eg demographic, clinical, social) and information on exposures and potential confounders	10-12	Table 1 reports demographic characteristics; Table 2 reports descriptive statistics and correlations for exposures/predictors. These include sex, age, education, job category, experience, IPC education, job stress, organizational culture, resource support, knowledge, and practice scores.
		(b) Indicate number of participants with missing data for each variable of interest	9	Variable-specific missing data counts were not reported. The manuscript only states that 3 questionnaires with insufficient responses were excluded before the final analysis.
		(c) *Cohort study*—Summarise follow-up time (eg, average and total amount)	N/A	Not applicable.
Outcome data	15*	*Cohort study*—Report numbers of outcome events or summary measures over time	N/A	Not applicable.
		Case-control study—Report numbers in each exposure category, or summary measures of exposure	N/A	Not applicable.
		Cross-sectional study—Report numbers of outcome events or summary measures	10-14	Cross-sectional outcome summary measures are reported as Foley and CVC IPC practice means/SDs, group comparisons, correlations, and mixed-model/mediation results (Tables 1-4).
Main results	16	(*a*) Give unadjusted estimates and, if applicable, confounder-adjusted estimates and their precision (eg, 95% confidence interval). Make clear which confounders were adjusted for and why they were included	12-14	Table 3 provides adjusted LMM estimates with standard errors, standardized coefficients, t values, and p values; Table 4 provides direct and indirect effects with 95% confidence intervals. Adjusted covariates are explicitly stated in the Table 3 note.
		(*b*) Report category boundaries when continuous variables were categorized	10-11	Category boundaries are reported in Table 1 for categorized variables such as age (<40, 40-49, 50-59, ≥60 years) and experience (<5, 5-<10, 10-<15, ≥15 years).
		(*c*) If relevant, consider translating estimates of relative risk into absolute risk for a meaningful time period	N/A	Not applicable.

Continued on next page

Other analyses	17	Report other analyses done—eg analyses of subgroups and interactions, and sensitivity analyses	10, 13-17	Additional analyses included bivariate correlations, bootstrap mediation analysis, and a sensitivity analysis additionally adjusted for facility-level IPC capacity (Supplementary Table S1).
**Discussion**				
Key results	18	Summarise key results with reference to study objectives	14, 18	The Discussion and Conclusions summarize the main findings in relation to the study objective.
Limitations	19	Discuss limitations of the study, taking into account sources of potential bias or imprecision. Discuss both direction and magnitude of any potential bias	18	The Limitations section discusses the cross-sectional design, self-reporting/reporting bias, low internal consistency for the CVC knowledge subscale, and the limited number of participating hospitals.
Interpretation	20	Give a cautious overall interpretation of results considering objectives, limitations, multiplicity of analyses, results from similar studies, and other relevant evidence	14-18	Interpretation is cautious and framed against prior literature, technical demands of device types, infrastructural constraints in LTCHs, and alternative explanations (e.g., possible suppression effect for job stress).
Generalisability	21	Discuss the generalisability (external validity) of the study results	18	Generalisability is addressed in the Limitation.
**Other information**				
Funding	22	Give the source of funding and the role of the funders for the present study and, if applicable, for the original study on which the present article is based	19	This work was supported by the National Research Foundation of Korea (NRF) … This funding source had no role in the study design, analysis, data interpretation, or decision to submit the manuscript for publication.

*Give information separately for cases and controls in case-control studies and, if applicable, for exposed and unexposed groups in cohort and cross-sectional studies.

Note: An Explanation and Elaboration article discusses each checklist item and gives methodological background and published examples of transparent reporting. The STROBE checklist is best used in conjunction with this article (freely available on the Web sites of PLoS Medicine at http://www.plosmedicine.org/, Annals of Internal Medicine at http://www.annals.org/, and Epidemiology at http://www.epidem.com/). Information on the STROBE Initiative is available at www.strobe-statement.org.
